# Response to mTOR and PI3K inhibitors in enzalutamide-resistant luminal androgen receptor triple-negative breast cancer patient-derived xenografts

**DOI:** 10.7150/thno.36182

**Published:** 2020-01-01

**Authors:** Florence Coussy, Marion Lavigne, Leanne de Koning, Rania El Botty, Fariba Nemati, Adnan Naguez, Guillaume Bataillon, Berengère Ouine, Ahmed Dahmani, Elodie Montaudon, Pierre Painsec, Sophie Chateau-Joubert, Fuhrmann Laetitia, Thibaut Larcher, Sophie Vacher, Walid Chemlali, Adrien Briaux, Samia Melaabi, Anne Vincent Salomon, Jean Marc Guinebretiere, Ivan Bieche, Elisabetta Marangoni

**Affiliations:** 1 Unit of Pharmacogenomics, Department of Genetics, Institut Curie, 26 rue d'Ulm, Paris, France; 2 Laboratory of Preclinical Investigation, Department of Translational Research, Institut Curie Research Center, 26 rue d' Ulm, Paris, France; 3 Department of Medical Oncology, Institut Curie, 26 rue d' Ulm, Paris, France; 4 Department of Biopathology, Institut Curie, 26 rue d' Ulm, Paris, France; 5 Translational Research Department, RPPA Platform, Institut Curie Research Center, 26 rue d'Ulm, Paris, France; 6 BioPôle Alfort, National Veterinary School of Alfort, Maison Alfort, France; 7 INRA, APEX-PAnTher, Oniris, Nantes, France; 8 Department of Biopathology, Institut Curie, Hopital René Huguenin, Saint Cloud, France; 9 U1016, Paris Descartes University, 4 avenue de l'observatoire, Paris, France.

**Keywords:** Triple-negative breast cancer, androgen receptor, luminal androgen receptor (LAR), genomic alteration, targeted therapy, PI3K pathway inhibitor.

## Abstract

Luminal androgen receptor (LAR) breast cancer accounts for 10% of all triple-negative breast cancers (TNBC). Anti-androgen therapy for this subtype is in development, but yields only partial clinical benefits. In this study, we aimed to characterize the genomic alterations of LAR TNBC, to analyze activation of the PI3K signaling pathway and to compare the response to PI3K pathway inhibitors with that to anti-androgen therapy in patient-derived xenografts (PDX) of LAR TNBC.

**Methods**: Four LAR PDX models were identified, on the basis of their transcriptomic profiles, in a cohort of 57 PDX models of TNBC. The expression of *AR*-related genes, basal and luminal cytokeratins and EMT genes was analyzed by RT-PCR and IHC. *AKT1* and *PIK3CA* mutations were identified by targeted NGS, and activation of the PI3K pathway was analyzed with a reverse-phase protein array. Three LAR PDXs with a *PIK3CA* or *AKT1* mutation were treated with the AR inhibitor enzalutamide, a PI3K inhibitor, a *dual* PI3K-mTOR inhibitor and a mTORC1-mTORC2 inhibitor. Finally, we screened a clinical cohort of 329 TNBC for *PIK3CA* and *AKT1* hotspot mutations.

**Results**: LAR TNBC PDXs were significantly enriched in *PIK3CA* and *AKT1* mutations, and had higher levels of luminal-androgen-like gene expression and a higher PI3K pathway protein activation score than other TNBC subtypes. Immunohistochemistry analysis revealed strong expression of the luminal cytokeratin CK18 and AR in three LAR PDX models. We found that mTOR and PI3K inhibitors had marked antitumor activity *in vivo* in PDX harboring genomic alterations of *PIK3CA* and *AKT1* genes that did not respond to the AR antagonist enzalutamide. *PIK3CA* mutations were detected in more than one third of AR+ TNBC from patients (38%), and only 10% of AR-negative TNBC.

**Conclusion**: Our results for PDX models of LAR TNBC resistant to enzalutamide indicate that *PIK3CA* and *AKT1* are potential therapeutic targets.

## Introduction

Triple-negative breast cancer (TNBC) accounts for about 10% of all breast cancers 1. This disease is defined by the absence of estrogen receptor (ER) and progesterone receptor (PR) expression, and of human epidermal growth factor receptor 2 (*ERBB2*) overexpression 2. TNBC generally has a poor prognosis, due to a lack of targeted therapy, in particular 3. TNBC are highly heterogeneous in terms of their genomic and histological characteristics, and this may have hampered efforts to develop effective treatments. Various studies have addressed the issue of the heterogeneity of gene expression in TNBC 4, 5, 6. Lehman et al. identified six classes of TNBC on the basis of transcriptomic analyses: basal-like1: BL1; basal-like2: BL2; immunomodulatory: IM; mesenchymal: M, mesenchymal stem-like: MSL; and luminal androgen receptor: LAR 5. Each of these classes was characterized by alterations to specific pathways. The LAR subtype is the most differentiated subtype of TNBC and it accounts for about 10% of all TNBC (9% in the METABRIC cohort and 8.7% in the TCGA cohort) 7, 8. This subtype is characterized by activation of the androgen receptor (AR) pathway, with expression of the genes encoding AR targets and coactivators. The prognosis of this subtype remains unclear, due in particular to differences in its classification on the basis of gene expression 7, 9 or histological descriptions 10. Anti-androgen therapy is currently being developed for this subtype and has been shown to have definite, but only partial clinical benefits 11, 12.

The phosphatidylinositol 3-kinase (PI3K) signaling pathway is crucial for cell growth and survival. *PIK3CA* activating mutations and *PTEN* loss of expression may contribute to treatment resistance in breast cancer (BC). The LAR subtype, associated with the luminal phenotype, is enriched in PI3K pathway alterations 13. However, no clinical data are available concerning the activity of PI3K inhibitors in this subtype.

PDX models are robust preclinical models for testing the suitability of genomic alterations for use as biomarkers and comparing responses to targeted therapy, as they conserve the molecular heterogeneity present in the patient 14 and are predictive of treatment response in clinical practice 15. However, no PDX models of LAR TNBC have ever been described, possibly due to the low frequency of this subtype of breast cancer.

The objective of this study was to characterize the genomic and protein characteristics of LAR PDXs and to compare the efficacy of various therapies targeting the PI3K signaling pathway with that of AR inhibitors.

## Materials and Methods

### Patients

We analysed samples from 323 unilateral invasive non-metastatic triple-negative primary breast tumors excised from women managed at Institut Curie (Paris and Saint-Cloud, France) between 1980 and 2015 (Table S1). Most of the patients (67%) were diagnosed and treated after 2000. All patients admitted to our institution before 2007 were informed that their tumor samples might be used for scientific purposes and were given the opportunity to refuse such use. Since 2007, patients admitted to our institution also provide consent actively, by signing an informed consent form. Patients (mean age: 56 years, range: 28-91) met the following criteria: primary unilateral non-metastatic TNBC, with full clinical, histological and laboratory data and full follow-up at Institut Curie. Median follow-up was 7.8 years (range: 8 months to 36 years). Eighty-one patients developed metastases within 10 years.

### Patient-derived xenografts

LAR PDX were identified in a recently described large cohort of TNBC PDX 16. Clinical information for the four LAR patients is provided in table S2. The experimental protocol and animal housing complied with institutional guidelines, and with the requirements of the French Ethics Committee (Agreement B75-05-18, France). Three LAR PDX models with specific alterations were chosen for *in vivo* preclinical assays: HBCx-2 (*AKT1* mutation), HBCx-31 (*AKT1* mutation), HBCx-154 (*PIK3CA* mutation). A fourth model, HBCx-35, was lost after five passages in mice and was not used for experiments. These three models were treated five times per week with enzalutamide (50 mg/kg, once daily), five times per week with PF-04691502 (10 mg/kg, once daily) (MedChem Express®), three times per week with BAY80-6946 (14 mg/kg) (MedChem Express®), and five times per week with AZD2014 (15 mg/kg) (MedChem Express®). Time of sacrifice depending on treatment: BAY80-6946: 3h post treatment, PF-04691502: 1h post treatment, AZD2014: 4h post treatment.

Tumor growth was evaluated by measuring two perpendicular tumor diameters with calipers, twice weekly. Individual tumor volumes were calculated as follows: V=axb2/2, where “a” is the largest diameter, and “b” is the smallest diameter. For each tumor, volume is expressed relative to the initial volume, as relative tumor volume (RTV). Tumor growth inhibition (TGI) on treatment was assessed by calculating the ratio of the mean RTV (relative tumor volume) for the treated group to the mean RTV for the control group at the same time point. The statistical significance of TGI was assessed in a paired Student's *t* test comparing tumor volumes between the treated and control groups. *p*-values were considered statistically significant for *p < 0.05, **p < 0.01 and *** *p* < 0.001.

### Transcriptomic data analysis

Transcriptomic profiling was performed with gene expression arrays on 57 PDX TNBC. The concentration and integrity/purity of each RNA sample were determined with the RNA 6000 LabChip kit (Agilent) and an Agilent 2100 bioanalyzer. Samples were hybridized with GeneChip Human 1.1 ST arrays in accordance with the manufacturer's (Affymetrix) recommendations, with the WT Expression Kit protocol (Life Technologies) and Affymetrix labeling and hybridization kits. The RMA normalization procedure was applied with the oligo package 17. No additional human-mouse cross-hybridization filtering was applied, as our xenograft samples contained less than 5% mouse cells (determined by RT-PCR to quantify transcripts of the ubiquitously expressed *TBP* gene with specific mouse and human primer pairs), too small a proportion to affect the expression profiles obtained with HuGene1.0 arrays 18. The TNBC molecular subtypes of the PDX were determined from gene expression data, with TNBCtype software developed by Chen et al. 19.

**Somatic mutation analysis:** We analyzed 57 triple-negative PDX by targeted NGS on 95 genes, selected from those most commonly mutated in breast cancer (>1%) and including potential therapeutic targets (Table S3). Specific NGS primers were designed based on the human reference genome (<1% of the total reads common to mice). NGS was performed on an Illumina HiSeq2500 sequencer. Reads were aligned with the BWA allowing up to 4% mismatches with the reference sequences. Only reads with a mapping quality of more than 20 were used for variant calling with the GATK unified genotyper. The genomic alterations detected included single-nucleotide variations of SMGs (i.e. base substitutions and short insertions/deletions) 20. Genomic variants were annotated with data from the 1000 Genome and COSMIC databases 21. Deleterious genomic alterations were defined as follows: (i) for oncogenes, we considered only mutations resulting in a gain of function (i.e. hotspot missense mutations, in-frame insertions/deletions/splicing variants reported to be oncogenic), (ii) for tumor suppressor genes (TSG), we considered only mutations resulting in a loss of function (i.e. biallelic truncating alterations (nonsense mutations, frameshift insertions/deletions/splicing) or monoallelic truncating alterations associated with heterozygous deletions detected by copy number analysis). Variants with a low allelic frequency (<5%) or low coverage (<100x) were excluded from the analysis. Genomic variants were validated biologically by comparison with the COSMIC, TumorPortal and cBioportal databases 6, 22.

### Analysis of copy number alterations (SCNA)

PDX were profiled with Affymetrix genomics arrays (SNP 6.0 or Cytoscan HD array). Genome-wide copy number analysis was performed with Affymetrix SNP arrays, as previously described 23, 24. SNP 6.0 or Cytoscan HD arrays were processed with 500 ng and 250 ng of gDNA, respectively, as the starting material, as recommended by the supplier. Raw data were normalized with Genotyping Console (SNP6.0 arrays) or Chromosome Analysis Suite (Cytoscan HD arrays). The focal amplification of oncogenes was defined as a log ratio>1.58 (6 copies per diploid genome) and a maximum size <10 megabases. Biallelic inactivation of TSG was defined as a homozygous deletion or truncating mutation associated with heterozygous deletion. Copy number alterations were compared with those of TCGA breast cancers in cBioPortal data 25, 26.

### Screening for *PIK3CA* and *AKT1* mutations in patients

*PIK3CA* mutations (exons 1, 2, 9, 20), and *AKT1* (E17K, hotspot) were detected by sequencing cDNA fragments obtained by RT-PCR amplification. The exons from the two genes to be screened were chosen on the basis of the mutation frequency reported in COSMIC: Catalogue of Somatic Mutations in Cancer (cancer.sanger.ac.uk/). Screening was performed by high-resolution melting curve analysis on a LightCycler 480 (Roche Diagnostics, Penzberg, Germany) with LCGreen Plus + Melting Dye fluorescence (Biotech, Idaho Technology Inc., Salt Lake City, UT). Details of the primers and PCR conditions are available on request. The amplified products were sequenced with the BigDye Terminator kit on an ABI Prism 3130 automatic DNA sequencer (Applied Biosystems, Courtaboeuf, France) with a detection sensitivity of 5% mutated cells, and the sequences were compared with the corresponding cDNA reference sequences (PIK3CA NM_006218, AKT1 NM_005163). All the mutations detected were confirmed in a second independent sample testing run.

### RT-qPCR in PDXs

Total RNA extraction and RT-PCR have been described elsewhere 27. Expression of the *TBP* gene (GenBank accession no. NM_003194) encoding the TATA box-binding protein (a component of the DNA-binding protein complex TFIID) was quantified as an endogenous RNA control and each sample was normalized against its TBP mRNA content. Results, expressed as N-fold differences in target gene expression relative to the TBP gene and termed “Ntarget”, were determined as Ntarget = 2ΔCt sample, where the ΔCt value of the sample was determined by subtracting the mean Ct value of the target gene from the mean Ct value of the TBP gene 28.

**Figure 1:**
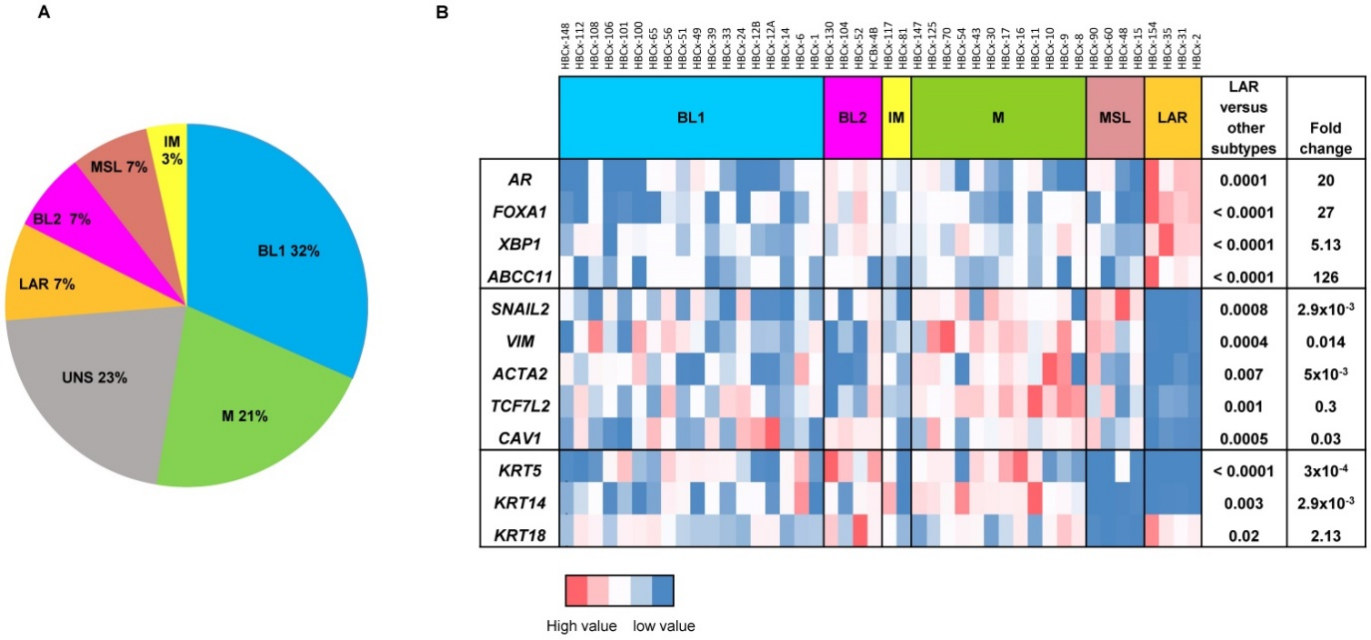
** Gene expression analysis of TNBC PDX. (A)** Classification of PDX according to Lehmann's classification of TNBC (N=57). BL1: Basal-like1; BL2: Basal-like 2; IM: Immuno-modulatory, M:Mesenchymal; MSL: Mesenchymal Stem Like; LAR: Luminal Androgen Receptor; UNS: Unstable); **(B)** RT-PCR expression analysis of AR- related genes (*AR, FOXA1, XBP1, ABCC11*) , EMT genes (*SNAIL2, VIM, ACTA2, TCF7L2, CAV1*) and cytokeratin's KRT5, KRT14, KRT18 in TNBC PDX (n=44). Fold changes and p value are calculated to analyze gene expression in LAR PDX as compared to the other TNBC subtypes.

For the analysis of gene expression in PDX, mRNA levels were normalized to obtain a 'basal mRNA level' (smallest amount of mRNA quantifiable (Ct = 35)) equal to 1. We analyzed the expression of AR-related genes (AR, FOXA1, XBP1, ABCC11), genes involved in epithelial-mesenchymal transition (EMT) (SNAI2, VIM, ACTA2, TCF7L2, CAV1) and three cytokeratin genes (KRT5, KRT14, KRT18) in all TNBC PDX. The 13 PDX classified as unstable (UNS) in Lehmann's classification were excluded from the RT-PCR analysis shown in Figure 1B. Mann-Whitney tests were used to analyze gene expression differences between LAR and the other TNBC subtypes. Fold-changes in expression were calculated from the ratio of the mean value for LAR to the mean value for the subtypes.

The expression of AR-related genes (*AR, FOXA1, PIP, TFAP2B*) and AR-induced genes* (FN1, SERPINB5, S100P)* was analyzed in treated xenografts.

### Reverse-phase protein arrays (RPPA)

RPPA was performed as previously described 29 for 48 of the 57 TNBC PDX (9 PDX were established after the RPPA analysis)**.** We calculated a PI3K pathway score with normalized data to assess pathway activation. Scores were obtained by calculating the sum for positive protein components (PI3K p110 subunit β, p-AKT1 (Ser473), p-AKT1 (Thr308), p-4E-BP1, p-p70-S6 kinase, p-S6 ribosomal protein; Cell Signaling Technology®) and subtracting the negative components of the pathway (PTEN, Cell Signaling Technology®). Eleven PDX classified as unstable (UNS) in Lehmann's classification were excluded from the analysis shown in Figure 3B.

### Western blot analysis

Proteins were extracted from tumors in RIPA buffer (50 mM Tris HCL pH 8, 150 mM NaCl, 0.5% deoxycholic acid, 0.5% Triton), supplemented with protease and phosphatase inhibitors. Lysates were resolved by electrophoresis in 10% agarose gels. The resulting bands were transferred onto nitrocellulose membranes (Bio-Rad, Hercules, CA, USA), which were then probed with rabbit antibodies against AKT, p-AKT (Ser473), S6, p-S6, 4E-BP1, p-4E-BP1, PRAS, p-PRAS and GAPDH (Cell Signaling®). The membranes were washed and incubated with the appropriate horseradish peroxidase-conjugated affinity-purified goat anti-rabbit secondary antibodies (Jackson ImmunoResearch Laboratories, Inc., Interchim). Protein was quantified with Multi Gauge software and normalized against GAPDH levels.

### Immunohistochemistry (IHC) analysis

TNBC PDX were fixed in 10% neutral buffered formalin, embedded in paraffin and stained with hematoxylin-eosin. TNBC PDX were included in TMAs in duplicate. Two cores were picked from each tumor paraffin block, with the Tissue-Tek Quick-Ray System from Sakura and a 6x10 matrix of the 2 mm core recipient block; 4 µm TMA sections were allowed to adhere to Superfrost Plus slides (MICROM, Walldorf, Germany). AR (Cell Signaling #5153, 1/400), CK5 (AbCam, ab52635, 1/400), CK8/18 (cloneE431-1, Invitrogen, 1/100), CK14 (Abcam, ab198167, 1/100), EGFR (Cell Signaling, #4267, 1/100), P-S6 (Cell Signaling ,#5364, 1/2000), and P-AKT (Ser 473,Cell Signaling, #4060, 1/50) antibodies were used. Slides incubated in parallel with pre-immune rabbit IgG were used as negative controls. Incubation and detection by the streptavidin-biotin-peroxidase complex method with DAB as the substrate were performed with a Ventana Medical System (ROCHE) DXT automat.

### Statistical analysis

The proportions of the TNBC subgroup transcriptome were compared in Chi^2^ tests. The proportions of genomic alterations between PDX and TCGA were compared in Chi^2^ or Fisher's exact tests, as appropriate.

Metastasis-free survival (MFS) was determined as the interval between diagnosis and detection of the first distant metastasis. Overall survival (OS) was determined as the interval between diagnosis and death. Survival distribution was estimated by the Kaplan-Meier method.

## Results

### Establishment and characterization of LAR TNBC PDXs

Four PDX models of the LAR TNBC subtype were identified from a recently reported cohort of 57 TNBC PDX 16. In this cohort, the Lehmann classification of TNBC subtypes was determined on the basis of transcriptomic profiles, with the TNBCtype tool. All Lehmanns' TNBC subtypes were represented: 32% BL1, 21% M, 7% LAR, 7% MSL, 7% BL2, 3% IM and 23% UNS (Figure 1A). We validated the correlation between transcriptomic classification and gene expression, by analyzing the expression of different sets of genes (*AR*-related genes, basal cytokeratin genes and EMT-related genes) by RT-PCR in 44 TNBC PDX (we excluded 13 PDX classified as unstable (UNS)). Figure 1B shows the heat map of normalized gene expression for the AR-related genes (*AR, FOXA1, XBP1, ABCC11*), cytokeratin genes (*KRT5, KRT14, KRT18*) and EMT genes (*SNAI2, VIM, ACTA2, TCF7L2,* and* CAV1*). The LAR subtype is characterized by significant overexpression of *AR*-related genes relative to the other TNBC subtypes. As expected, luminal cytokeratin *KRT18* was overexpressed and the basal cytokeratins (*KRT5, KRT14*) were significantly underexpressed in the LAR subtype. Various genes associated with epithelial-mesenchymal transition (EMT: *SNAI2, VIM, ACTA2, TCF7L2, and CAV1*) were underexpressed in LAR PDX relative to the other TNBC subtypes.

**Figure 2:**
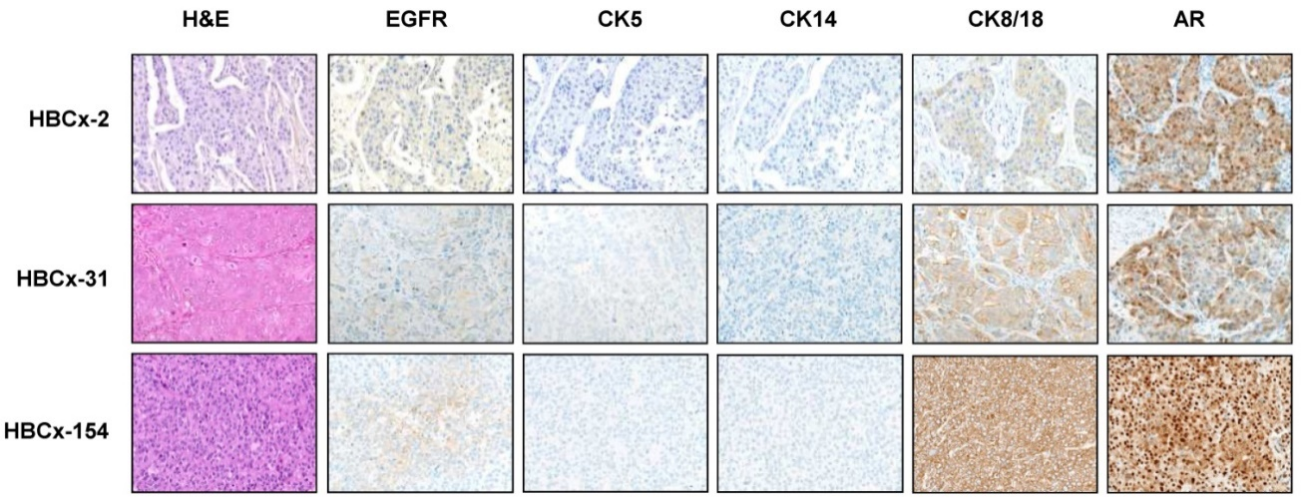
** Morphological and immunohistochemistry analysis of 3 LAR TNBC PDX.** Representative hematoxylin-eosin (H&E) stained sections and expression of EGFR, CK5, CK14, CK8/18 and AR (20X).

LAR TNBC are characterized by an apocrine morphology 30. We therefore analyzed the histology of the LAR TNBC PDXs. Three cases, shown in Figure 2, presented typical apocrine differentiation. Tumor cells presented the characteristic nuclear and cytoplasmic features that must be present in more than 90% of tumor cells to confirm the morphological diagnosis of apocrine differentiation: (i) abundant (nuclear-cytoplasmic ratio = 1:2 or greater), finely granular eosinophilic cytoplasm (ii) round or pleomorphic nuclei (iii) prominent red nucleoli, often multiple (iv) sharply defined cell borders. On immunochemistry, tumor cells are usually positive for AR. AR was strongly expressed in HBCx-154 PDX (90%, +++), whereas weaker nuclear expression was observed in the HBCx-2 and HBCx-31 PDX. The three PDXs expressing the luminal cytokeratin CK18 and were negative for basal cytokeratins (CK5, CK14).

### The PI3K signaling pathway is activated in the LAR subtype

**Figure 3:**
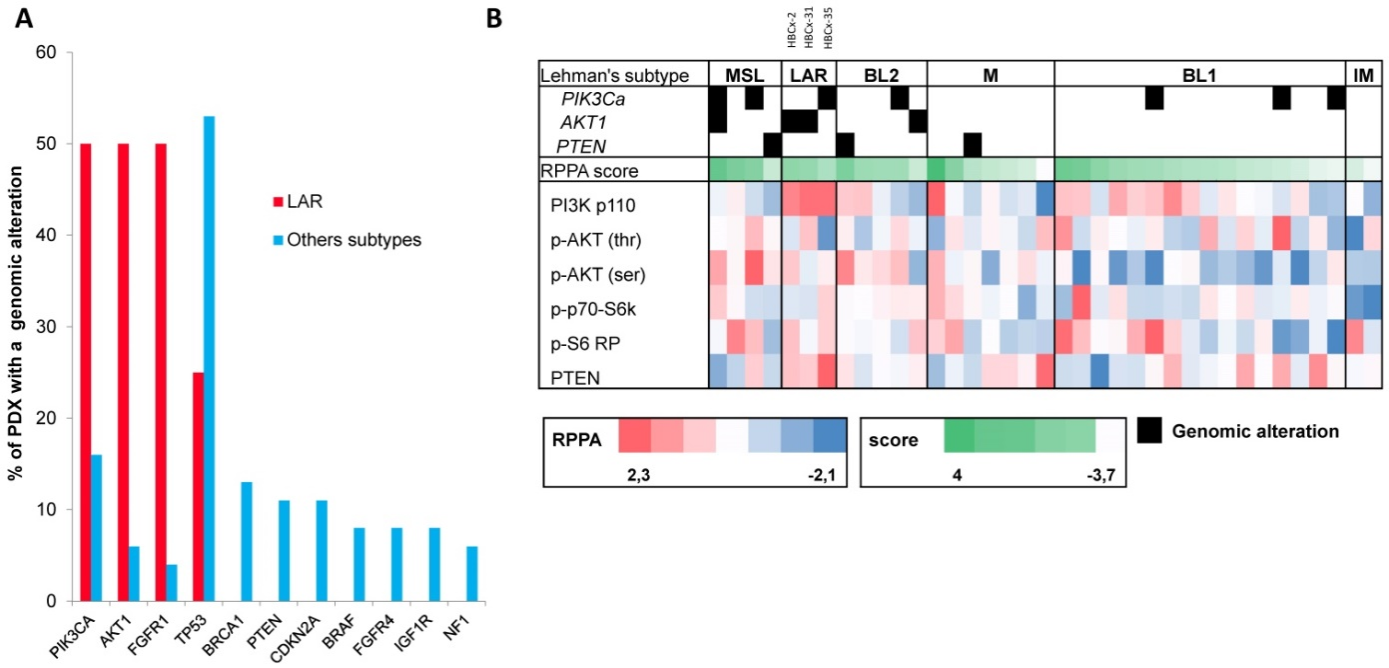
** Genomic alterations and activation of PIK3/AKT pathway in LAR PDX as compared to the other TNBC subtypes. (A)** Percentage of genomic alterations (mutations, amplification and homozygous deletions) in TNBC PDX. **(B)** Heatmap representing *PIK3CA, AKT1* and *PTEN* genomic alterations and expression of PI3Kp110, P-AKT, P-p70-S6K, P-S6 RP and PTEN proteins determined by RPPA analysis in the 37 TNBC PDX. RPPA activation score was determined by calculating the sum of the different protein components. (3 LAR models =HBCx-2, HBCx-31, HBCx-35)

LAR TNBC are characterized by a high frequency of *PIK3CA* mutations 13. We therefore compared the genomic profile of LAR PDX (*n*=4) with that of the other TNBC subtypes. According to targeted NGS (analysis of the 95 genes most frequently altered in breast cancer) and copy number analysis, LAR TNBC PDX had a genomic profile different from that of the other TNBC subtypes, with enrichment in *PIK3CA* and *AKT1* mutations (100% versus 7,5%, *p*=0.0002, Fisher's exact test; LAR versus other subtypes), a lower frequency of *TP53* mutations (25% for LAR and 52.8% in the other subtypes) and a high frequency of *FGFR1* amplification. LAR models were also characterized by a lower frequency of genomic alterations in genes associated with the cycle cell pathway and an absence of alterations to the MAPK and DNA repair pathways (Table 1) (Figure 3A).

**Figure 4:**
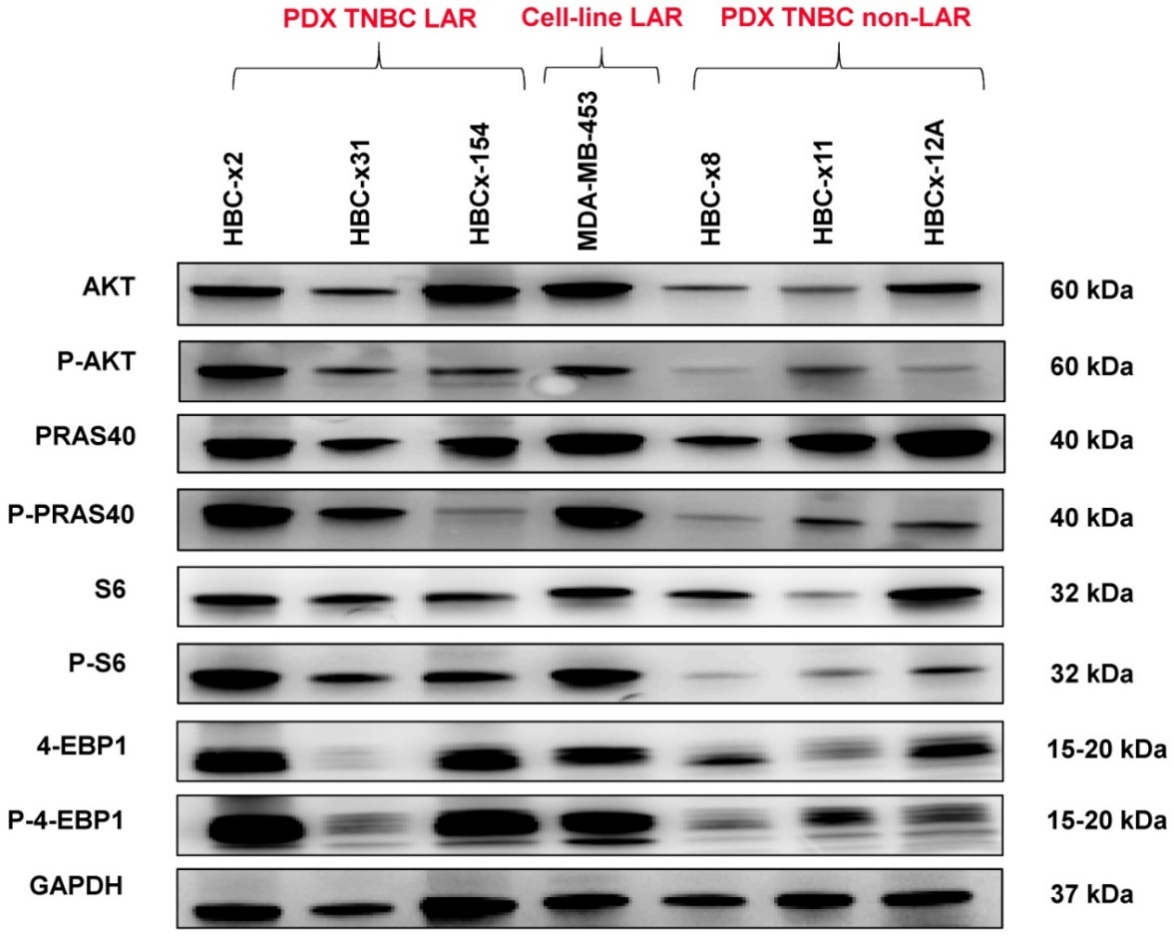
Western Blot analysis of different PI3K/AKT/mTOR pathway components in the 3 LAR PDX, a LAR cell line (MDA-MB 453) and 3 non LAR TNBC PDX models.

We analyzed the expression of the major effectors of the PI3K pathway (PI3-kinase, p-AKT, p-4E-BP1, p70-S6-kinase, p-S6RP, PTEN) by RPPA, to confirm the activation of this signalling pathway. The MSL and LAR subtypes had higher PI3K pathway activation scores: MSL TNBC are driven by low levels of PTEN protein and LAR TNBC, like other luminal breast cancers, are driven by high levels of PI3K protein (Figure 3B). A western blot analysis of P-AKT, PRAS, P-S6 and P 4EBP1 confirmed the activation of the PIK3 pathway in the three LAR TNBC PDX. Three TNBC PDX (HBCx-8, HBCx-11, HBCx-12A) with low levels of AR mRNA were used as negative controls (Figure 4 and S1) (mean Ct/TBP Hs vs. 35 for the 3 LAR PDX were 1776 versus 0.25 for the 3 negative controls).

### Efficacy of PI3K pathway inhibitors in 3 LAR TNBC PDX

**Figure 5:**
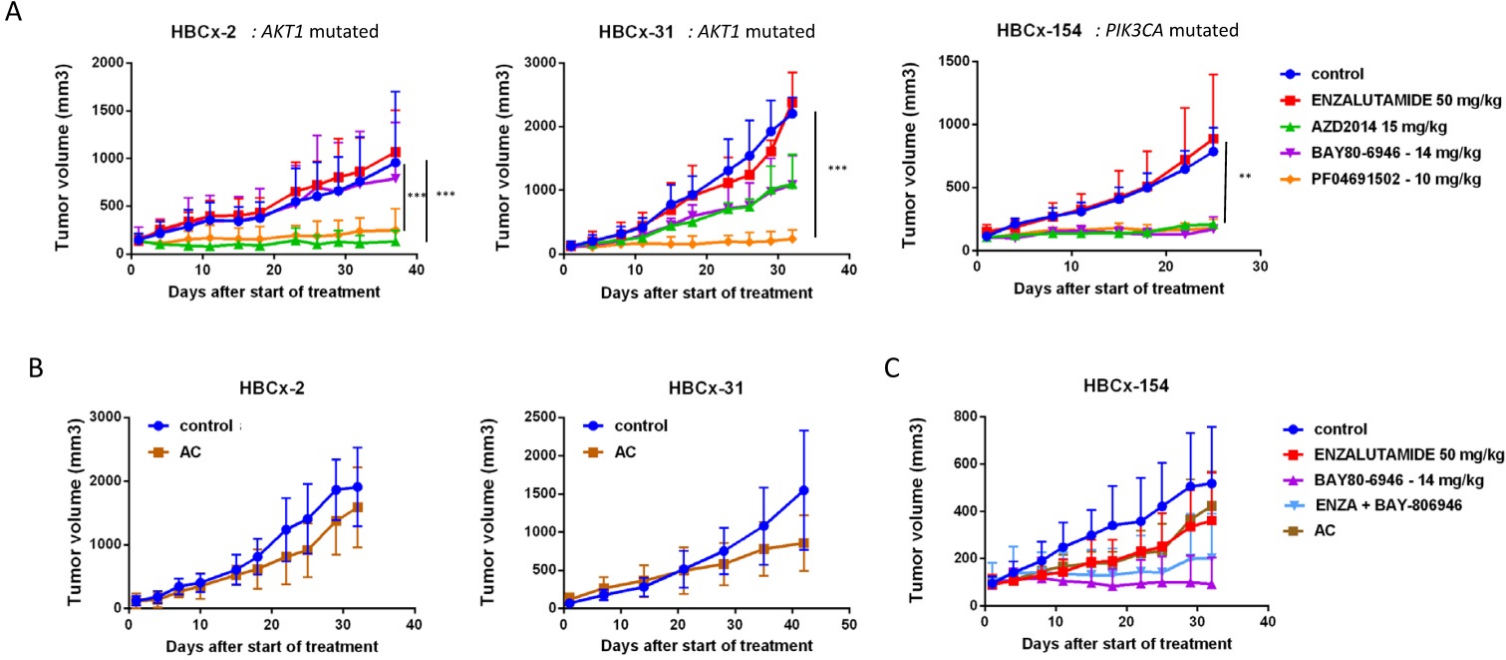
***In vivo* response to targeted therapies and chemotherapy in LAR PDX.** (A) *In vivo* response to enzalutamide, AZD2014 (dual mTORC1 and C2 inhibitor), BAY80-6946 (PI3K inhibitor) , PF-04691502 (dual PI3K and mTOR) in the 3 LAR PDX. mean +/- SD. (B) Response to AC (Adriamycin + cyclophosphamide) in the HBCx-2 and HBCx-31 PDX. (C) response to AC and to the combination of BAY80-6946 + enzalutamide in the HBCx-154 PDX.

We assessed the dependence of LAR PDX tumor growth on AR signaling by the *in vivo* efficacy of enzalutamide, an androgen receptor inhibitor approved for the treatment of prostatic cancer. Surprisingly, enzalutamide was completely ineffective in the three PDX (TGI:-28% for HBCx-2 and 27% for HBCx-31 and HBCx-154) (Figure 5). We investigated the possible association between enzalutamide treatment and a decrease in AR-targeted gene expression, by analysing the expression of various AR-related genes and AR-inducible genes in control and enzalutamide-treated xenografts, by RT-PCR. The expression of AR-related and AR-inducible genes in treated xenografts was similar to that in controls (Figure S2).

LAR TNBC PDX are characterized by *AKT1* or *PIK3CA* mutations (associated with the activation of PI3K protein signaling). We therefore compared three different PI3K pathway inhibitors (BAY80-6946, a specific inhibitor of the PI3K p110α subunit; PF-04691502, a dual inhibitor of PI3K and mTOR; and AZD2014, an mTORC1 and mTORC2 inhibitor) with enzalutamide. HBCx-2 and HBCx-31 harbored an *AKT1* hotspot mutation (p.E17K), whereas HBCx-154 harbored two *PIK3CA* mutations (p.N345K and p.H1048L). In the *PIK3CA* mutant model (HBCx-154), treatment with the three PI3K pathway inhibitors resulted in a significant inhibition of tumor growth (TGI= 80% for BAY-80-6946, *p*=0.006, 72% for PF-04691502 and AZD2014, *p*=0.02 and *p*=0.01, respectively). PI3K pathway inhibitors did not decrease tumor growth in the two *AKT1*-mutated models, but HBCx-2 PDX responded to the mTOR inhibitor AZD2014 (TGI=86%, *p*=0.0001) and the dual inhibitor (mTOR and PIK3Ca) (TGI =70%, *p*=0.001). The second *AKT1*-mutated model (HBCx-31) presented a significant response only to the dual inhibitor (TGI=81%, *p*=0.02). We also tested a combination of adriamycin and cyclophosphamide [AC] (standard chemotherapy used in breast cancer treatment) in the three models: all three PDX were resistant to AC (Figure 5 B and C). Previous studies in cell line models of LAR breast cancer have reported increased antitumor activity of PI3K inhibitors used in combination with AR inhibitors 13. We therefore tested the combination of BAY-80-6946 and enzalutamide in the HBCx-154 PDX, the *PIK3CA-*mutated PDX with the strongest nuclear AR expression. Combination treatment resulted in no greater antitumor activity than observed with BAY-80-6946 alone (Figure 5C).

**Figure 6:**
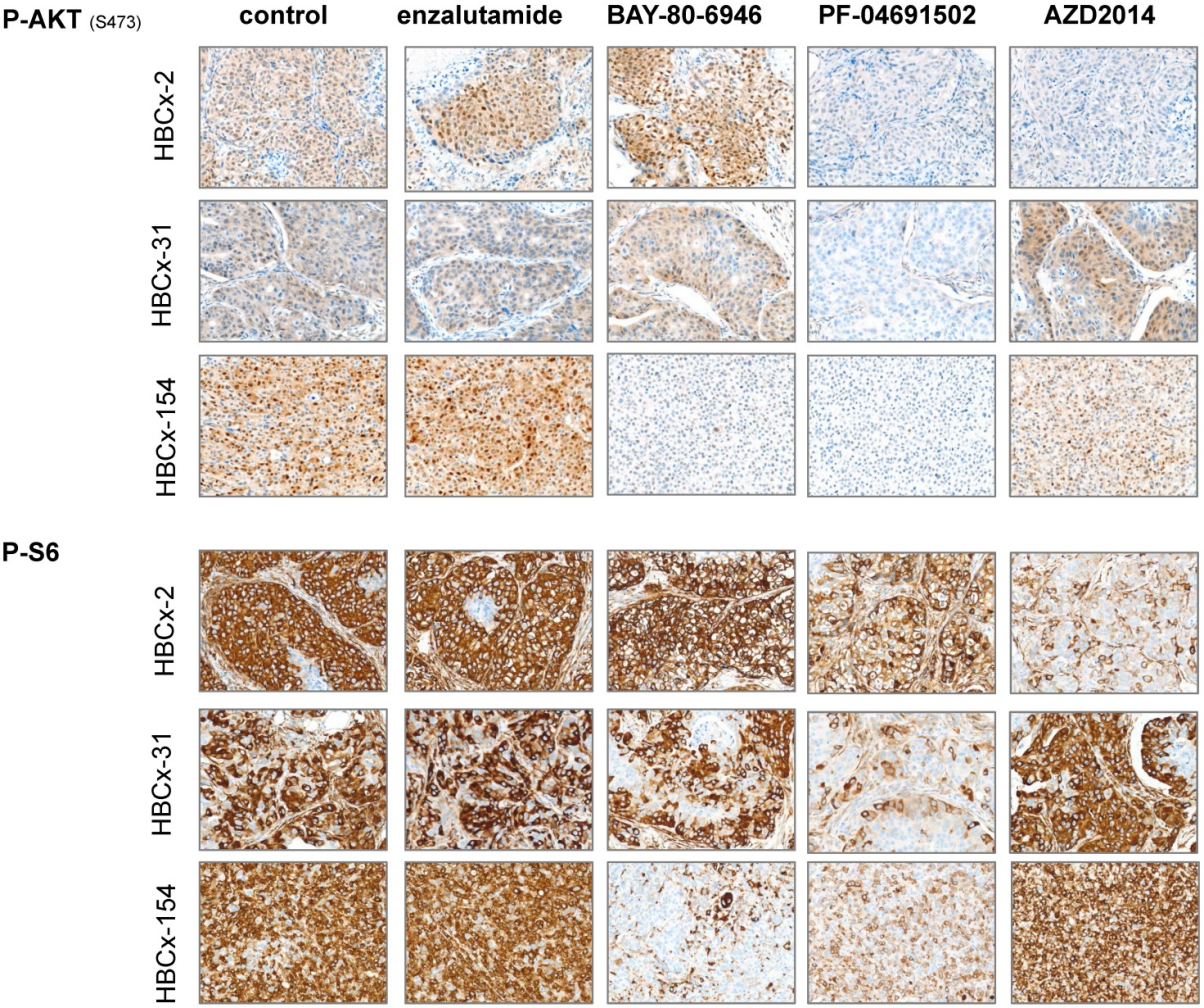
** Immunohistochemistry analysis of 3 LAR TNBC PDX:** Analysis of p-AKT and p-S6 before and after treatments with enzalutamide, BAY-80-6946, PF-04691502 and AZD2014 (X20)

We analyzed pathway inhibition in treated tumors, by performing an IHC analysis of P-AKT and P-S6 on tumors harvested from control and treated xenografts. We confirmed the lower levels of P-AKT and P-S6 expression (i) in the 3 models after PF-04691502 treatment, (ii) in HBCx-154 after BAY-80-6946 treatment and (iii) in HBCx-2 after AZD2014 treatment (Figure 6).

### Prognostic value of *PIK3Ca* mutation

We analyzed *PIK3CA* and *AKT1* hotspot mutations in a large cohort of TNBC with no distant metastasis at diagnosis, treated at the Institut Curie, to confirm the high frequency of *PIK3CA* and *AKT1* mutations in AR+ TNBC tumors. Transcriptomic analysis is not routinely performed in clinical practice. We therefore focused on 21 TNBC positive by IHC for AR (AR+)(>10%) from a cohort of 323 TNBC. All of these cases were characterized by an apocrine morphology. *PIK3CA* mutations were strongly correlated with AR+ TNBC (38% (8/21) versus 10% (30/302) in other subtypes; Chi^2^ =0.001), but *AKT1* mutations were not correlated with AR+ TNBC (4.7% (1/21) versus 3.3% in other subtypes, Chi^2^ =0.5) probably due to the small size of the sample of tumors presenting these alterations. On univariate analysis, *PIK3CA* mutations were associated with a poorer prognosis in the overall population of TNBC, for both metastasis-free survival (MFS, *p*=0.01) and overall survival (OS, *p*=0.0016) (Figure S3), but these associations were not significant on multivariate analysis. Details of the prognostic factors identified are provided in Table S4. In the population of patients with apocrine tumors, the association was close to significance, but the small number of patients made it impossible to draw robust conclusions (*n*=21; MFS: *p*=0.06 and OS: *p*=0.07) (Figure S3).

## Discussion

Luminal androgen receptor-positive (LAR) tumors are a rare subtype of TNBC, accounting for 9% of our TNBC PDX cohort 7, 8. LAR is the most highly differentiated subtype of TNBC. Gene ontology analyses reveal an enrichment of LAR TNBC in hormonally regulated pathways. Lehmann et al. found that* AR* mRNA levels were much higher in LAR TNBC than in the other TNBC subtypes, and that tumors of the LAR group expressed numerous downstream AR targets and coactivators 7. Our findings confirm the strong expression of AR-related genes in the LAR subtype, associated with low levels of EMT gene expression (these genes being more strongly expressed by the M and MSL subtypes), consistent with the luminal properties of these tumors.

The role of AR in carcinogenesis has been studied more extensively in prostate cancer, in which there is compelling evidence for a crucial role of this pathway, due to its effects on prostate cell proliferation and differentiation. Furthermore, in clinical practice, androgen inhibitors constitute the cornerstone of medical treatment for prostatic cancer 32. The role of AR in breast cancer is complex and depends on the signaling pathways activated simultaneously 33. The lack of efficient targeted therapies and the poor prognosis of TNBC has led to the development of AR-targeted therapies in TNBC with AR expression.

Moreover, chemoresistance is much more frequent in LAR than in the other subtypes, as demonstrated by Masuda, who reported a low rate of pCR after primary chemotherapy in LAR TNBC 34, highlighting the need for new therapies for this subtype of TNBC.

Little is known about the genomic features of the LAR subtype. In the TCGA cohort, LAR TNBC displayed a higher proportion of *PIK3CA* mutations than other subtypes (46.2% versus 4.5%, *p*<0.0001) 5. *PIK3CA* mutations are associated with AR protein levels 35. We report specific features of this subtype in terms of genomic alterations and the activation of signaling pathways. Mutations of the *PIK3CA* and *AKT1* genes are frequent in LAR PDX (100%; 4/4 PDX models) and AR+ TNBC (38%; 8/21 patients for *PIK3CA*). The PIK3 pathway is the only major pathway altered in the LAR subtype, contrasting with the situation in other subtypes and opening up new possibilities for effective treatment. As in luminal breast cancer, this activation involves *PIK3C*A and *AKT1* mutations to a greater extent than *PTEN* loss (which is more frequently reported in TNBC in general). No specific data are available concerning the levels of PI3K pathway proteins in the LAR subtype. An analysis of the proteins present in 105 breast cancers from the TCGA cohort confirmed that PI3K was expressed in luminal A and B breast cancers, but also in TNBC 36. We analyzed the activation of the PI3K pathway in the various TNBC subtypes, confirming its correlation with genomic alterations.

The aim of this study was, therefore, to compare PI3K pathway inhibitors with an AR antagonist in chemoresistant LAR PDX models. We describe here the first LAR PDX models used to test the efficacy of targeted therapies in this TNBC subtype. Indeed, few data are available for LAR models: only cell lines or CDX (cell line-derived xenografts) have been described 5, 13.

Our three LAR PDX models were resistant to the AR inhibitor enzalutamide and to standard chemotherapy for breast cancer (combination of anthracyclines and cyclophosphamide). Lehman et al. described the effects of the AR inhibitor bicalutamide in LAR cell lines 13. Three clinical trials have tested androgen inhibitors in TNBC and have reported a clinical benefit of 19% to 29% 11, 12. The Advanced Breast Cancer (ABC4) guidelines propose androgen inhibitors as a treatment option for AR^+^ TNBC 37. Despite the potential utility of AR inhibitors in the treatment of LAR tumors, the clinical benefits of such treatment are modest and unmet clinical needs remain for LAR TNBC.

Our PDX models displaying PI3K pathway activation responded strongly to PI3K inhibitors, suggesting that *PI3KCA* and *AKT1* mutations drove the proliferation of these tumors. As expected, BAY-80-6946, a specific inhibitor of PI3K, was effective in the *PIK3CA*-mutated model, but not in the two *AKT1*-mutated PDX 38. The PI3K-mTOR dual inhibitor (PF-04691502) strongly decreased tumor growth in the three LAR PDXs. This dual inhibitor abolished the PI3K pathway more completely, by inhibiting all catalytic isoforms of PI3K as well as mTORC1 and mTORC2 39. AZD2014 was effective in two models (one with a *PIK3CA* mutation and one with a *AKT1* mutation). AZD2014 inhibited mTORC1 and mTORC2 and abolished the feedback loops related to the inhibition of mTORC1 alone, thereby increasing the efficacy of a specific mTORC1 inhibitor 40. In HBCx-154 PDX (*PIK3CA*-mutated) the 3 compounds had similar anti-tumor activities, however inhibition of P-AKT and P-S6 was higher in xenografts treated by BAY-80-6946 and PF-04691502 compounds, suggesting that in this tumor inhibition of PI3K is necessary to inhibit PI3K-Akt signaling pathway.

Despite the lack of enzalutamide activity, we hypothesized that a combination of enzalutamide and a PI3K inhibitor would promote tumor regression, as a synergistic effect has been described in an LAR cell line 13. However, the combination of enzalutamide and BAY80-6946 in the* PIK3CA*-mutated PDX model with the highest levels of AR expression did not result in greater antitumor activity than BAY80-6946 monotherapy. This finding is not consistent with Lehmann's results showing a pan-PI3K inhibitor and a dual PI3K/mTOR inhibitor to be more effective in LAR CDX than bicalutamide in two cell-line models: bicalutamide slowed growth at the limit of significance, but the PI3K inhibitor appeared to be more effective both alone and in combination with bicalutamide 13, and several clinical trials of combination therapy for AR^+^ TNBC are currently underway (NCT02457910, NCT03207529).

Our LAR models present primary resistance to enzalutamide, which can be explained partly by the low levels of nuclear AR expression in the HBCx-2 and HBCx-31 models, although the AR present seems to be active, as shown by the high levels of AR-related gene expression. The presence of oncogenic activating mutations associated with PI3K pathway activation and the sensitivity to PIK3CA/mTOR inhibitors observed suggest that *AKT1* and *PIK3CA* mutations are the main drivers of cell proliferation in these tumors. Alternatively, enzalutamide may not target AR in these models, as suggested by the absence of inhibition of AR-dependent gene expression after enzalutamide therapy. However, the dose used in our study (50 mg/kg/day) has been shown to be effective in the cell line-derived xenograft model of ER breast cancer 41 and in xenograft models of prostate cancer 42. Moreover, we can speculate that the engraftment of androgen-dependent TNBC may be compromised in female nude mice, in which androgen production may be insufficient to support AR-dependent tumor growth. Our results show that the combination of PI3K and AR inhibitors does not increase sensitivity. Reciprocal feedback inhibition of AR by PI3K signaling in prostatic cancer has been reported, with the inhibition of PI3K leading to the derepression and activation of AR target genes, and the inhibition of AR leading to reciprocal PI3K pathway activation 43. However, in the HBCx-154 model, PI3K pathway inhibition did not result in changes in AR-related gene expression (data not shown). PI3K inhibition thus appears to be insufficient to reverse AR resistance, but other mechanisms of resistance have been reported, including NFkB activation, *MYC* gain, and mitochondrial reprogramming 44. Further investigations, possibly involving enzalutamide-resistant TNBC cell lines, are required, to investigate the molecular mechanisms underlying the crosstalk between PIK3 signaling and enzalutamide resistance.

In clinical practice, AKT1 inhibitors will probably be developed for the treatment of tumors of the LAR subtype, and interesting results have already been obtained for combinations of these agents with chemotherapy in TNBC 45.

The prognostic impact of AR in TNBC patients remains a matter of debate. Lehmann described a tendency towards a poorer prognosis for LAR (distant metastasis-free survival) 7. By contrast, Masuda reported a tendency towards better survival for LAR after primary chemotherapy 34. In a meta-analysis of TNBC patients, AR expression was also found to be associated with a better prognosis 35. Furthermore, in breast cancer, *PIK3CA* mutation is significantly associated with longer invasive disease-free survival (*p* = 0.043), but not with longer distant DFS or OS 46. The prognostic value of these parameters appears to have been demonstrated more clearly for estrogen receptor-positive breast cancer 47. Multivariate analysis of our TNBC cohort did not confirm the prognostic value of *PIK3C*A mutation.

In conclusion, our results provide a robust rationale for screening for *PIK3CA* and* AKT1* mutations in AR-TNBC patients and for treating selected patients with PI3K/AKT/mTOR pathway inhibitors.

## Supplementary Material

Supplementary figures and tables.

## Figures and Tables

**Table 1:**
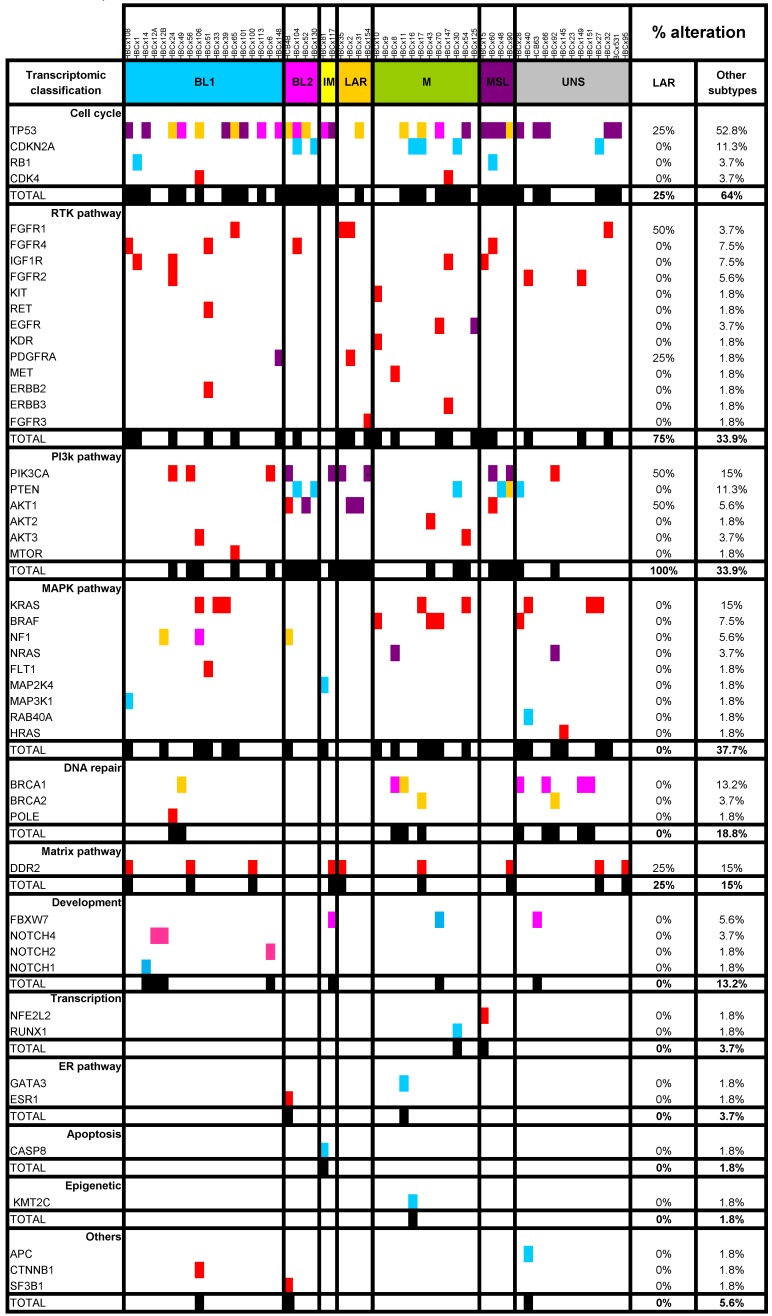

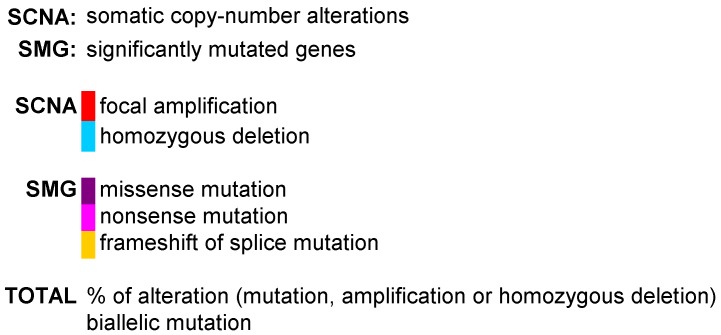
Representation of SMGs and SCNAs for 57 TNBC PDXs

## Abbreviations

AR: Androgen receptor
BL1: Basal-like1
BL2: Basal-like2
CDX: Cell line-derived xenografts
CK: Cytokeratin
EMT: Epithelial-mesenchymal transition
ER: Estrogen receptor
ERBB2: Human epidermal growth factor receptor 2
IM: Immunomodulatory
LAR: Luminal androgen receptor
M: Mesenchymal
MFS: Metastasis-free survival
MSL: Mesenchymal stem-Like
OS: Overall survival
PDX: Patient-derived xenografts
PI3K: Phosphatidylinositol 3-kinase
PR: Progesterone receptor
RPPA: Reverse-phase protein arrays
RTV: Relative tumor volume
TGI: Tumor growth inhibition
TNBC: Triple-negative breast cancers
