# Supplementary material for: Response to mTOR and PI3K inhibitors in enzalutamide-resistant luminal androgen receptor triple-negative breast cancer patient-derived xenografts
Source: Theranostics. 2020 Jan 1;10(4):1531–43. doi: 10.7150/thno.36182 (PMC6993232; doi:10.7150/thno.36182)
Supplement: Supplementary file 1 — Supplementary figures and tables. [file thnov10p1531s1.pdf]

Figure S1: **Analysis of P-AKT, P-PRAS, P-S6 and P-4EBP1 expression in 3 LAR PDX (HBCx-2, HBCx-31, HBCx-154) , LAR cell line (MDA-MB-453) and 3 non LAR PDX (HBCx-8, HBCx-11, HBCx-12A)**
Graphs represent protein expression (phospho-protein/total protein) determined by western blot analyzed.

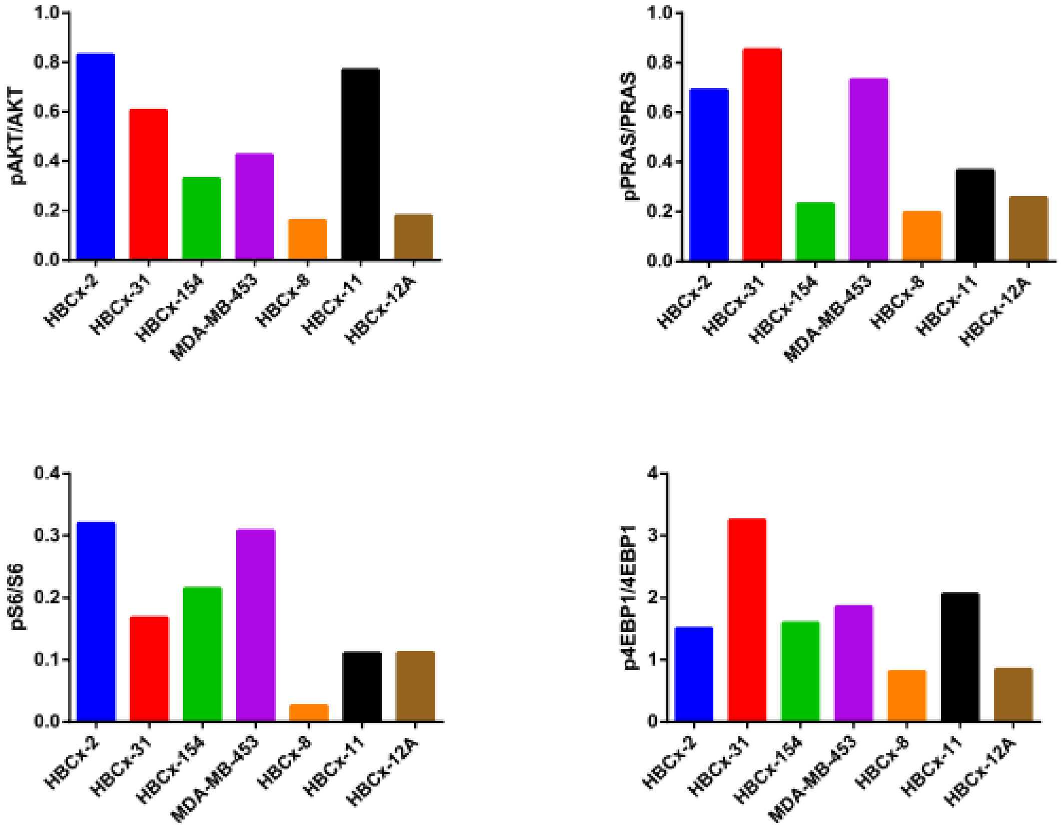

Figure S2: RT-PCR analysis of the expression of AR-related genes (*AR, FOXA1, PIP, TFAP2B*), AR-inducible genes (*FN1, SERPINE5, S100P*) before (CRL) and after treatment with enzalutamide. Data are normalized by Log2 transformation

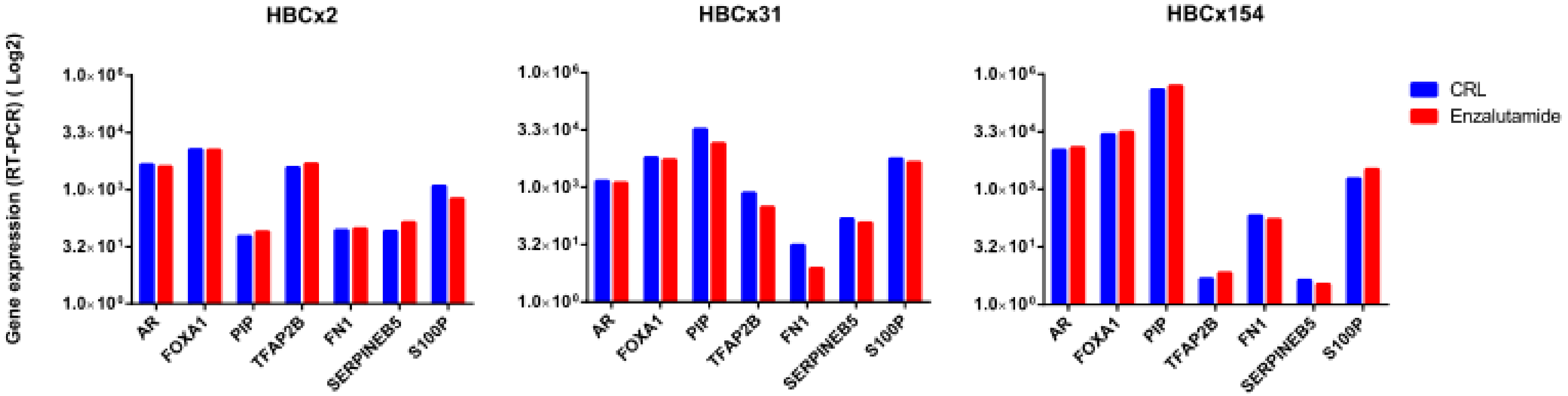

Figure S3: Metastasis-free survival at 10 years (MFS) and overall survival at 10 years (OS) in the TNBC population (n=323) (upper panel) and in the AR+ TNBC population (*n*=21) (lower panel)

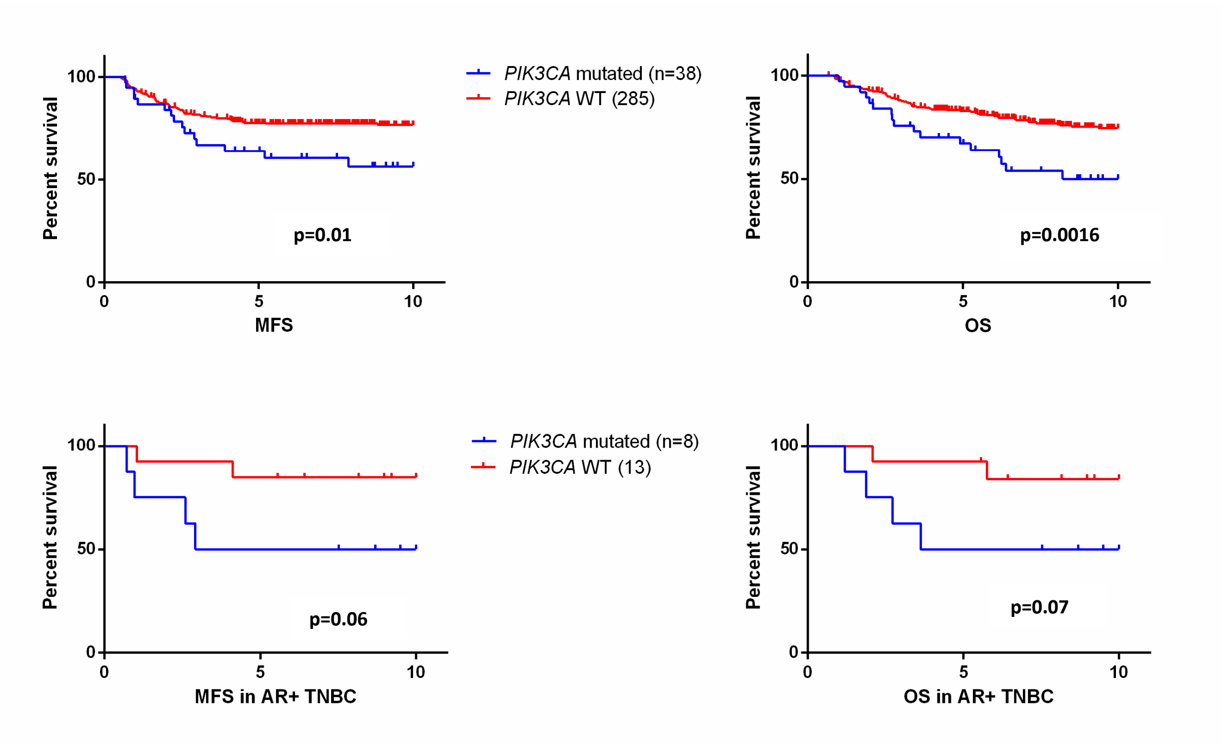

**Mean age** in years (range)		56 (28-91)
		n (%)
**T (TNM)**	1	115 (35.6)
	2	194 (60)
	3	14 (4.4)
**N (TNM)**	0	200 (62)
	1	79 (25)
	2	30 (9)
	3	12 (4)
**SBR**	1	5 (1.6)
	2	41 (13)
	3	270 (85.4)
**Chemotherapy**	Yes	246 (84.5)
	No	74 (25.5)
**Radiotherapy**	Yes	242 (83.2)
	No	49 (16.8)
**Subtype**	NST	220 (68.2)
	Medullary	36 (11.1)
	Rare	33 (10.2)
	Apocrine	21 (6.5)
	Metaplastic	13 (4)
**PIK3CA status**	WT	285 (88.2)
	Mutated	38 (11.8)
**AKT1 status**	WT	312 (96.6)
	Mutated	11 (3.4)

Table S1: Characteristics of the 323 TNBC patients

Table S2: Clinical and genomic characteristics of the LAR PDX models

PDX	Type of graft	Age at diagnosis	T	N	Breast surgery	Node surgery	Histologic type	LVI	Grade SBR	RO	RP	HER2	Chemotherapy	RT	*PIK3CA* status*	*AKT* status*	Other genomic alterations*	MFS (months)	Distant relapse (o:no; 1:yes)	OS (months)	Status 0=alive;1=dead
HBCx-154	Primary breast cancer	83	1	1	Mastectomy	Lymphadenectomy	NST	-	3	-	-	-	No	No	M(p.N345K; p.I	WT	FGFR3 amplification	24	0	24	0
HBCx-31	Primary breast cancer	44	2	1	Mastectomy	Lymphadenectomy	Apocrine	+	3	-	-	-	(anthracycline	Yes	WT	M (p.E17K)	TP53 (p.R175H)	8	0	8	0
HBCx-35	Primary breast cancer	63	2	1	Mastectomy	Lymphadenectomy	NST	-	3	-	-	-	No	No	M (p.H1047R)	WT	FGFR1 and DDR2 amplification	25	0	25	0
HBCx-2	Primary breast cancer	76	2	1	Mastectomy	Lymphadenectomy	NST	+	3	-	-	-	No	Yes	WT	M (p.E17K)	FGFR1 and PDGFRA amplification	11	1	15	1

NST No special type
RT Radiotherapy
LVI Lymphovascular invasion
* Genomic analysis of the PDX

Table S3: List of genes tested in NGS

PATHWAY	GENES	NM
**Apoptosis**	APAF1	181861.1
	CASP8	001228.4
**Cell Cycle**	CDK4	000075.3
	CDK6	001259.6
	CDKN2A	000077.4
	P27/CDKN1B	004064.4
	PPP2R1A	014225.5
	PTPN11	002834.3
	RB1	000321.2
	STAG2	006603.4
	TP53BP1	005657.2
	TP73	005427.3
	TP53	000546.4
**Development**	FBXW7	033632.3
	NOTCH1	017617.3
	NOTCH2	024408.3
	NOTCH4	004557.3
**DNA repair**	BRCA1	007294.3
	BRCA2	000059.3
	POLE	006231.2
**Epigenetic**	ARID1A	006015.4
	CHD4	001273.2
	CREBBP	004380.2
	CTCF	006565.3
	MLL/ KMT2A	005933.3
	MLL2/KMT2D	003482.3
	MLL3/ KMT2C	170606.2
	TBL1XR1	024665.4
**ER pathway**	AR	000044.3
	ESR1	000125.3
	FOXA1	004496.3
	GATA3	002051,2
	NCOR1	006311.3
	PGR	000926.4
**MAPK pathway**	ALK	004304.4
	BRAF	004333.4
	DDR2	006182.2
	FLT1	002019.4
	HRAS	005343.2
	NRAS	002524.4
	KRAS	004985.4
	MAP2K1	002755.3
	MAP2K4	003010.3
	MAP3K1	005921.1
	NF1	000267.3
	RAB40A	080879.2
	ROS1	002944.2

**RTK pathway**	IGF1R	000875.4
	EGFR	005228.3
	ERBB2	004448,3
	ERBB3	001982.3
	ERBB4	005235.2
	FGFR1	023110.2
	FGFR2	000141.4
	FGFR3	000142.4
	FGFR4	002011.4
	KIT	000222.2
	MET	000245.2
	RET	020975.4
	KDR	002253.2
	PDGFRA	006206.4
**Matrice pathway**	CDH1	004360.3
	LAMA2	000426.3
	LAMA4	002290.4
	THBS1	003246.2
**PI3K-Akt-mTOR**	AKT1	005163.2
	AKT2	001626.5
	AKT3	005465.4
	INPP4B	003866.2
	MTOR	004958.3
	PIK3CA	006218.2
	PIK3R1	181523.2
	PTEN	000314.4
	STK11	000455.4
	TSC1	000368.4
	TSC2	000548.3
**Transcription**	CBFB	001755.2
	HIST1H3B	003537.3
	MALAT1	NR_002819.2
	MED1	004774.3
	MYB	005375.2
	NFE2L2	006164.3
	RUNX1	001754.4
	SETD2	014159.6
	TBX3	016569.3
**Others**	AGTR2	000686.4
	APC	000038.5
	CTNNB1	001904.3
	KEAP1	012289.3
	LDLRAP1	015627.2
	MYH9	002473.4
	MYO3A	017433.4
	SF3B1	012433.2
	STMN2	007029.3
	VHL	000551.3

Table S4: Univariate and multivariate (log-rank tests) analysis of metastasis-free survival (MFS) and overall survival (OS) at 10 years in 323 triple-negative breast cancers

	Number of patients	Number of distant metastases (%)	Log-rank test (univariate) MFS	Log-rank test (multivariate) MFS	Number of Death (%)	Log-rank test (univariate) OS	Log-rank test (multivariate) OS
**Years**							
<50	117	28 (24%)			28 (24%)		
>50	206	53 (25%)	0.3	NR	57 (27.6%)	0.17	NR
**T (TNM)**							
T1	115	19 (16.5%)			20 (17.4%)		
T2	194	56 (28.8%)			59 (30.4%)		
T3	14	6 (4.2 %)	**0.0034**	**0.03**	6 (42.8%)	**0.0055**	0.026
**N (TNM)**							
N0	200	33 (16.5%)			32 (16%)		
N1	79	25 (31.6%)			28 (35.4%)		
N2	30	14 (46.6 %)			16 (53.3%)		
N3	12	9 (75%)	**< 0.0001**	**< 0.0001**	9 (75%)	**< 0.0001**	**< 0.0001**
**Grade SBR**							
SBR1	5	1 (20 %)			1 (20%)		
SBR2	41	13 (31.7%)			14 (34.1%)		
SBR3	270	66 (24.4%)	0.9	NR	67 (24.8%)	0.7358	NR
**Radiotherapy**							
Yes	242	64 (26.5%)			65 (26.8%)		
No	49	15 (30%)	0.37	NR	14 (28.6%)	0.6424	NR
**Chemotherapy**							
Yes	246	56 (22.7%)			57 (23.1%)		
No	74	23 (31%)	0.07	NR	26 (35.1%)	**0.0203**	0.07
**Subtype**							
Apocrine	21	6 (28.5%)			6 (28.5%)		
Metaplastic	13	5 (38%)			5 (38.4%)		
Medullary	36	2 (5.5 %)			5 (13.8%)		
NST	220	59 (26.8%)			57 (26%)		
Rare	33	9 (27 %)	**0.046**	**0.0023**	12 (36.3%)	**0.0493**	**0.012**
***PIK3Ca* mutation**							
PIK3CA mutated	38	15 (39.4 %)			17 (44.7%)		
PIK3CA WT	285	66 (23.1 %)	**0.01**	0.39	63 (22%)	**0.0016**	0.24

NR: not relevant
